# Integration of Urinary Peptidome and Fecal Microbiome to Explore Patient Clustering in Chronic Kidney Disease

**DOI:** 10.3390/proteomes12020011

**Published:** 2024-04-01

**Authors:** Emmanouil Mavrogeorgis, Sophie Valkenburg, Justyna Siwy, Agnieszka Latosinska, Griet Glorieux, Harald Mischak, Joachim Jankowski

**Affiliations:** 1 Mosaiques Diagnostics GmbH, 30659 Hannover, Germany; mavrogeorgis@mosaiques.de (E.M.); siwy@mosaiques-diagnostics.com (J.S.); latosinska@mosaiques-diagnostics.com (A.L.); mischak@mosaiques.de (H.M.); 2 Institute for Molecular Cardiovascular Research (IMCAR), RWTH Aachen University Hospital, 52074 Aachen, Germany; 3 Nephrology Unit, Department of Internal Medicine and Pediatrics, Ghent University Hospital, 9000 Ghent, Belgium; sophie.valkenburg@uzgent.be (S.V.); griet.glorieux@ugent.be (G.G.); 4 Experimental Vascular Pathology, Cardiovascular Research Institute Maastricht (CARIM), University of Maastricht, 6229 Maastricht, The Netherlands

**Keywords:** chronic kidney disease, CE-MS, CKD, feces, microbiome, peptidome, urine

## Abstract

Millions of people worldwide currently suffer from chronic kidney disease (CKD), requiring kidney replacement therapy at the end stage. Endeavors to better understand CKD pathophysiology from an omics perspective have revealed major molecular players in several sample sources. Focusing on non-invasive sources, gut microbial communities appear to be disturbed in CKD, while numerous human urinary peptides are also dysregulated. Nevertheless, studies often focus on isolated omics techniques, thus potentially missing the complementary pathophysiological information that multidisciplinary approaches could provide. To this end, human urinary peptidome was analyzed and integrated with clinical and fecal microbiome (16S sequencing) data collected from 110 Non-CKD or CKD individuals (Early, Moderate, or Advanced CKD stage) that were not undergoing dialysis. Participants were visualized in a three-dimensional space using different combinations of clinical and molecular data. The most impactful clinical variables to discriminate patient groups in the reduced dataspace were, among others, serum urea, haemoglobin, total blood protein, urinary albumin, urinary erythrocytes, blood pressure, cholesterol measures, body mass index, Bristol stool score, and smoking; relevant variables were also microbial taxa, including *Roseburi*a, *Butyricicoccus*, *Flavonifractor*, *Burkholderiales*, *Holdemania*, *Synergistaceae*, *Enterorhabdus*, and *Senegalimassilia*; urinary peptidome fragments were predominantly derived from proteins of collagen origin; among the non-collagen parental proteins were FXYD2, MGP, FGA, APOA1, and CD99. The urinary peptidome appeared to capture substantial variation in the CKD context. Integrating clinical and molecular data contributed to an improved cohort separation compared to clinical data alone, indicating, once again, the added value of this combined information in clinical practice.

## 1. Introduction

Chronic kidney disease (CKD) progressively reduces kidney function in about one-tenth of the global population [1]. The comorbidities [2] and associated high costs [3,4,5] are evident in this condition. Standard clinical signs include consistent abnormalities in terms of structure or clinical markers (estimated glomerular filtration rate (eGFR) and albuminuria) for a minimum of three months [6]. Nevertheless, molecular changes may be indicative of disease pathophysiology, even before the manifestation of standard clinical signs. Determining the relevant molecular players might be crucial for shedding light on the disease mechanism. 

Along these lines, clinical samples collected through non-invasive methods, such as feces (microbiome) and urine (peptidome), seem promising for CKD research. Microbiota in the human gut [7] provide vital benefits to the host through their symbiotic relationship [8]. In that context, the health consequences associated with a dysregulated microbiota (‘dysbiosis’) may be well justified. Microbial changes have been identified to occur not only during kidney failure [9], but also in the earlier stages of CKD [10]. At the same time, the physiological disturbance in CKD extends to the proteome, which, with its various proteoforms, is associated with the biological complexity and phenotype of an individual. These pathophysiological changes can be reflected by aberrant protein fragment profiles observed in urine [11], which have been linked, among others, to CKD and CKD progression [12]. Along these lines, microbiome-based interventions, including probiotics [13], prebiotics [14], and fecal microbiota transplantation [15], are investigated for their effect on CKD, while urinary peptidome has also been part of a CKD clinical trial [16]. 

Nevertheless, despite this promising cumulative literature, a relevant unified multidisciplinary approach in the CKD context has not been performed yet. Therefore, in this study, the fecal microbiome [17] as well as the urinary peptidome (acquired by capillary electrophoresis coupled to mass spectrometry (CE-MS [11])) were collected from 110 CKD and Non-CKD individuals. In addition to the omics data, a variety of clinical information was compiled. The aim was to assess the capacity of each dataset (clinical and/or omics data, solely or in combinations) in separating the cohort according to CKD disease state (Non-CKD, Early, Moderate, Advanced). A series of preprocessing steps enabled the implementation of both numeric and nominal variables in the analyses. Using the different datasets, participants were clustered into four groups, and each time, the respective clustering quality was evaluated. Last, the impact of each clinical and molecular variable on the reduced dataspace was examined in an attempt to highlight the disease pathophysiology further.

## 2. Materials and Methods

### 2.1. Study Population

This study was based on 110 Non-CKD and CKD individuals recruited at the Ghent University Hospital [17]. In stages 1 to 5, there were, respectively, 12, 20, 40, 19, and 9 patients with CKD, along with 10 Non-CKD-diagnosed individuals. The eGFR was calculated using the Chronic Kidney Disease Epidemiology Collaboration (CKD-EPI) equation [18] and was used to determine the participants’ CKD stage. Based on these stages, the cohort was grouped into Non-CKD as well as Early (stages 1–2, eGFR ≥ 60 mL/min/1.73 m^2^), Moderate (stage 3, 30 mL/min/1.73 m^2^ ≤ eGFR < 60 mL/min/1.73 m^2^), and Advanced CKD (stages 4–5, eGFR < 30 mL/min/1.73 m^2^). Medication data were expressed as a 2nd level (pharmacological or therapeutic subgroup) of the anatomical therapeutic chemical (ATC) classification system [19].

Nevertheless, kidney function is generally found to be associated with, e.g., body mass index (BMI), age, blood pressure, etc., and as such, in this cohort, the four disease state groups differed in their clinical characteristics (Table 1A). Nevertheless, such differences may potentially impact the molecular profiles. That said, although fecal microbiome data were not available, the urinary peptidome data of a historical cohort were also considered in an attempt to account for the impact of clinical confounders. That cohort (n = 408) consisted of four equally numbered groups matched for the clinical variables described in Table 1B, namely, age, BMI, sex, as well as systolic and diastolic blood pressure. The matched individuals were derived from already-published studies [16,20,21,22,23,24,25,26,27,28,29,30,31,32,33,34,35,36]. The requirements per group were the same as before, with the exception of the Non-CKD group (eGFR ≥ 90 mL/min/1.73 m^2^ and, if known, urinary albumin-to-creatinine ratio (UACR) < 30 mg/g and absence of kidney disease) and the Early CKD group (eGFR ≥ 60 mL/min/1.73 m^2^ and UACR ≥ 30 mg/g). 

Of note, regarding the original cohort, the mean eGFR of the Non-CKD group appeared to be lower than that of the Early CKD group. This might be attributed, on the one hand, to a few of the Νon-CKD participants having an eGFR < 70 mL/min/1.73 m^2^, likely, at least to a degree, related to age; and, on the other hand, to the fact that the Early CKD patients were diagnosed considering additional characteristics on top of eGFR. 

### 2.2. Data Acquisition and Evaluation

The fecal microbiome and (most of the) clinical data originated from Gryp et al., 2021 [17], with the respective methods being described in the original publication. In the context of the STRATEGY-CKD consortium, the human urinary peptidome was acquired based on CE-MS [11] and is provided as Supplementary Table S1. A detailed technical description has already been published in original articles, such as in [37]. In brief, the urinary peptidome analysis was divided into three steps: sample preparation, CE-MS measurement, and data evaluation. Initially, 700 μL thawed urine aliquots were mixed with 700 μL of 2 M urea and 10 mM NH4OH containing 0.02% sodium dodecyl sulfate to suppress protein interactions. Subsequently, peptides and small proteins with molecular weight < 20 kDa were isolated with a Centrisart centrifugal filter device (Sartorius, Göttingen, Germany). A PD 10 gel filtration column (GE Healthcare Bio Sciences, Uppsala, Sweden) was utilized to clear away the urea, salts, and electrolytes from the obtained filtrate. Then, lyophilization and storage at 4 °C until the time of the CE-MS measurement followed, at which time samples were re-suspended in 10 μL HPLC-grade H_2_O. The CE relied on a P/ACE MDQ system (Beckman Coulter, Fullerton, CA, USA) that was connected to an electrospray ionization interface (Agilent Technologies, Palo Alto, CA, USA) with a potential of −4.0 kV to −4.5 kV, which produced ions towards a MicrOTOF II MS (Bruker Daltonics, Bremen, Germany) mass spectrometer. The running buffer consisted of 20% acetonitrile (Sigma-Aldrich, Taufkirchen, Germany) in HPLC-grade water (Roth, Karlsruhe, Germany) supplemented with 0.94% formic acid (Sigma-Aldrich). Sample injection volume into CE-MS was ~290 nL at 2 psi for 99 sec. Separation was performed at 35 °C, applying +25 kV voltage at the injection (capillary) side for 30 min. At the same time, pressure was applied at 0.1, 0.2, 0.3, and 0.4 psi for 1 min each, and lastly, at 0.5 psi for 30 min. The *m*/*z* spectrum range was 250–2500 (accumulation: every 3 s for about 80 min). The raw MS data evaluation was performed using MosaFinder software (version 1.4) [11]. Internal standards were used to calibrate the mass [Da] and CE migration time [min] based on global and local regression, respectively. In order to account for variation in sample concentration, an intensity-normalization step was considered, utilizing 29 disease-independent peptides (from housekeeping proteins) as internal standards. In that way, comparability across different datasets was achieved.

### 2.3. Data Preprocessing

In this study, clinical variables were considered in the subsequent analyses. Variables utilized in defining CKD stages, particularly those linked to the CKD-EPI formula [18], such as eGFR, age, sex, and serum creatinine (or related variables like smoking period and urinary creatinine), had been previously excluded to prevent potential bias. Only the urinary peptides with known sequence information were considered for further analyses. The microbial amplicon sequence variants (ASVs), obtained through 16S rRNA sequencing, were merged (summed) based on the microbial taxa information. Subsequently, data from both omics approaches were independently turned into sample proportions. Then, several preprocessing steps were applied. Initially, predefined frequency thresholds were set for each type of omics data. Specifically, a 30% frequency threshold was applied to the urinary peptidome, while a more tolerant 15% was applied to the microbiome data, given their high sparsity. Clinical variables were kept intact; triglycerides had the lowest percentage of non-missing values (30.9%). Subsequently, any variable with near-zero variance (percentage of unique values to total samples < 10% and ratio of most frequent to second most frequent value > 19) was removed. Missing data were imputed based on the k-nearest neighbor algorithm (clinical and microbiome variables; neighbors = 5) or the respective minimum value (peptidome variables). Categorical variables were also encoded into one or more binary columns. Then, the remaining data were normalized based on the ordered quantile normalization transformation [38] (as an attempt to turn skewed distributions into symmetric ones, potentially) and z-standardization (mean of zero and standard deviation of one) methods. The remaining processed variables were then used for the generation of datasets with the following information: (A) clinical, (B) microbiome, (C) clinical and microbiome, (D) peptidome, (E) clinical and peptidome, (F) microbiome and peptidome, and last, (G) clinical and microbiome and peptidome.

### 2.4. Dimensionality Reduction and Clustering

Each dataset was used as the basis for a partial least square (PLS) feature extraction. Variables were collectively transformed into a reduced space of three new features (PLS components). These transformed features were the product of maximizing, on the one hand, the variation in the original variables and, on the other, the relationship between them and the disease state variable (Early, Moderate, Advanced, or Non-CKD). In that way, the PLS components allowed for visualizing the participants as single data points in the 3D space. In an attempt to objectively assess how well these formations of participants were in line with distinct disease states, clustering around k = 4 medoids [39] was applied to the reduced dataspace. The contents of the generated clusters in terms of the disease state groups as well as the corresponding silhouette values were investigated. Last, the association of each variable with the reduced dataspace was assessed, with the rationale that the most impactful variables may as well be connected with the disease pathophysiology. 

### 2.5. Software

The data preprocessing and statistical analyses were based on R programming (R version 4.3.3) [40], running on Ubuntu 22.04 computer software. In that context, the ‘tidyverse’ collection of R packages (version 2.0.0) [41] as well as the ‘tidymodels’ package (version 1.1.1) [42] based on its very detailed resources [43] were used, among others. The PLS feature extraction was also performed using the ‘mixOmics’ package (version 6.24.0) [44]. The package ‘plotly’ (version 4.10.3) [45] was utilized for plotting the PLS components in the 3D space. The ‘patchwork’ (version 1.1.3) [46] package was used to illustrate the confusion matrices and clustering-related metrics in the same figure. The clustering of the data around medoids with the respective silhouette values was performed using the ‘cluster’ package (version 2.1.6) [47]. Matching was heavily based on the MatchIt package (version 4.5.5) [48]. Reproducibility can be achieved using set.seed (2020) before the relevant functions.

## 3. Results

**Figure 1:**
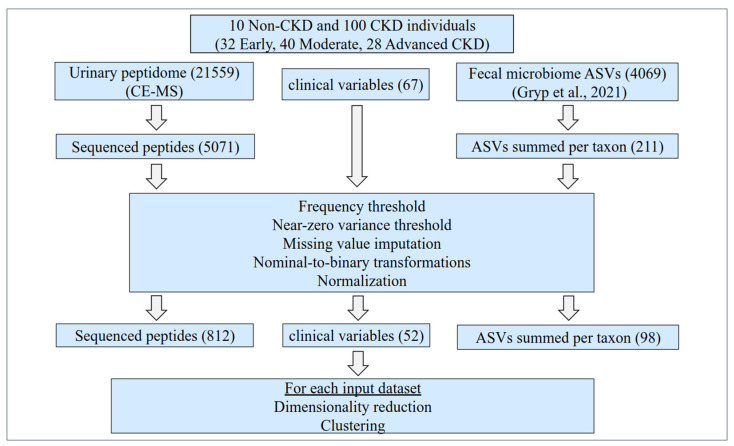
Study design. Initially, the clinical data, along with the fecal microbiome (Gryp et al., 2021) [17] and the human urinary peptidome (current study, acquired by CE-MS), were collected from 110 Non-CKD and CKD individuals. The participants were recruited at the Ghent University Hospital across all CKD stages, not on dialysis. For this study, 67 clinical variables, 211 summed ASVs according to microbial taxa, and 5071 sequenced urinary peptides were considered for further analysis. Predefined frequency and variance thresholds were applied. Missing values were imputed based on the k-nearest neighbor algorithm (clinical and microbiome variables) as well as the respective minimum value (peptidome variables). Subsequently, nominal variables were transformed to binary, and the 962 variables that remained were normalized. Each time, these clinical and/or molecular variables, alone or in combinations, were used as an input for a partial least square (PLS) feature extraction using the disease state as the target variable. The three generated PLS components were then used as the basis for k-medoids clustering (k = 4), and each time the capacity of each dataset for producing clusters in line with the disease state was evaluated based on participant visualizations in the reduced dataspace, the cluster contents, and the corresponding silhouette values. Of note, the urinary peptidome data of a historical, matched cohort (n = 408) from previously published studies [16,20,21,22,23,24,25,26,27,28,29,30,31,32,33,34,35,36] were also used for exploring the participants’ positioning in the reduced dataspace. ASVs: amplicon sequence variants; CE-MS: capillary electrophoresis coupled to mass spectrometry.

In this study, clinical, fecal microbiome, and urinary peptidome data corresponding to 110 CKD and Non-CKD individuals recruited at the Ghent University Hospital were studied. After a series of preprocessing steps, the remaining normalized 52 clinical, 98 microbiome, and 812 peptidome variables were considered for further analysis. The original cohort’s main clinical characteristics are described in Table 1A, while those of the matched historical cohort, in Table 1B. The study design is illustrated in Figure 1.

### 3.1. Cohort Visualizations in the 3D Space

The preprocessed variables of each dataset were transformed into three new features, namely, PLS components, and these were then used to visualize the individuals as single data points in low-dimensional space. In that way, the cohort’s formation based on disease state was visually examined per dataset. These visualizations are provided in 3D format as Supplementary Figure S1A–G.

Along these lines, shared patterns between the plots were demonstrated by our analyses. First, the tendency for separation that was observed in the plots between the different disease states was in line with the CKD group stage continuity, i.e., sequentially, Non-CKD, Early, Moderate, and Advanced CKD. Further, the Non-CKD group was relatively more distant and localized than the CKD groups. The microbiome plot per se was rather an exception to these observations, with generally overlapping and dispersed groups. Noteworthy is that, through the plots, several outlier individuals were positioned towards disease state groups with major differences in eGFR.

At the same time, dataset-specific patterns also existed in our analyses. A substantial overlap of the Moderate with both the Early and Advanced CKD groups was observed in the clinical plot (Supplementary Figure S1A). In the microbiome plot, the groups generally displayed higher overlap as well as a more balanced dispersion (Supplementary Figure S1B). Nevertheless, combining the clinical and microbiome datasets led to an improved separation of the Non-CKD group and clearer borders between the Advanced and the (still) overlapping Early and Moderate CKD groups (Supplementary Figure S1C). Further, combining both the peptidome and microbiome datasets with the clinical dataset, the overlap between the Early and Moderate CKD groups was almost diminished (Supplementary Figure S1G). Collectively, the latter formation of participants was similar to the one observed in plots generated using the peptidome alone or in combination with either the clinical or the microbiome data (Supplementary Figure S1D–F).

### 3.2. Clustering

**Figure 2:**
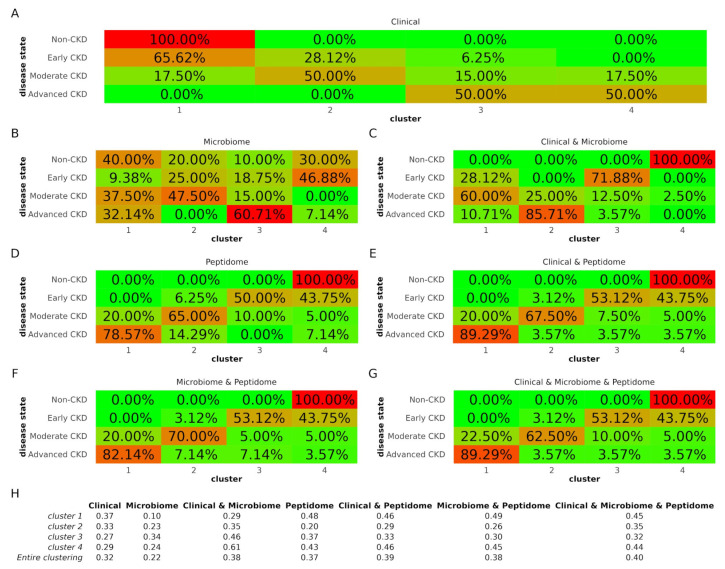
Clustering results. (**A**–**G**) Concordance between cluster content and disease state indicated by colors (green is low concordance and red is high concordance). Percentages refer to the proportion of the different disease state groups that were assigned to each cluster. (**H**) Silhouette values. Higher silhouette values indicate that members of the same clusters are well matched compared to neighboring clusters, while lower values suggest relatively poor matches. CKD: chronic kidney disease.

Each time, the PLS features were used as a basis for unsupervised clustering towards four groups. The contents of the generated clusters were then investigated; the assignment of just one disease state per cluster was considered ideal clustering, corresponding to a dataset of maximum capacity for cohort separation. The silhouette values were determined as an additional quality metric for the entire clustering procedure as well as per individual cluster. The clustering results are described in Figure 2. Of note, cluster numbers are independent between the different datasets.

Using the sole clinical dataset, cluster 1 contained mainly Non-CKD and Early CKD individuals, cluster 2 Moderate and Early CKD patients, and cluster 4 predominantly Advanced but also several Moderate CKD patients, while cluster 3 was a mixture of all but the Non-CKD group (Figure 2A). Compared to the clinical dataset, the microbiome dataset demonstrated highly mixed clusters (Figure 2B), while combining the clinical and microbiome datasets increased cluster “purity” (Figure 2C). The clusters of the peptidome dataset (Figure 2D) were more distinct compared to the microbiome, and the inclusion of clinical (Figure 2E) or microbiome data (Figure 2F) slightly increased the cluster purity. The clusters in the clinical and peptidome dataset were identical to those of the clinical, microbiome, and peptidome dataset (Figure 2G) regarding the distribution of all but the Moderate CKD group.

Considering the entire clustering, the silhouette values were in line with the results above regarding cluster “purity” (Figure 2H). In detail, the silhouette value for the clustering of the clinical dataset was 0.32, higher than that of the microbiome dataset (0.22). Nevertheless, when the clinical and microbiome datasets were combined, the silhouette value increased to 0.38. Peptidome clustering demonstrated a silhouette value of 0.37. This value was further boosted by the inclusion of the clinical (0.39), microbiome (0.38), or both datasets (0.40).

### 3.3. Variable Associations with the Transformed Dataspace

**Figure 3:**
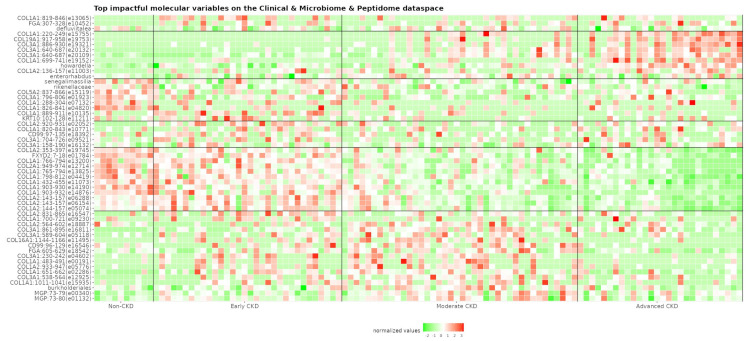
Heatmap of the 20 most impactful molecular variables per PLS component regarding the clinical, microbiome, and peptidome dataset. Variables of the urinary peptidome are labeled with their parental protein name and corresponding amino acid position (peptide ID in parenthesis), while those of the fecal microbiome are labeled using the relevant taxa information. Values (green: low, red: high) belong to the preprocessed dataset right before the dimensionality reduction step. The respective peptide sequences are described in Supplementary Table S1. CD99: CD99 antigen; COL16A1: collagen alpha-1(XVI) chain; COL19A1: collagen alpha-1(XIX) chain; COL1A1: collagen alpha-1(I) chain; COL1A2: collagen alpha-2(I) chain; COL2A1: collagen alpha-1(II) chain; COL3A1: collagen alpha-1(III) chain; COL5A2: collagen alpha-2(V) chain; FGA: fibrinogen alpha chain; FXYD2: sodium/potassium-transporting ATPase subunit gamma; KRT10: keratin, type I cytoskeletal 10; MGP: matrix Gla protein.

In an attempt to explore a potential link with disease pathophysiology, we explored which variables appeared to have the largest impact on the transformed dataspace (Supplementary Figure S2). The top 20 molecular variables per PLS component of the combined clinical, microbiome, and peptidome datasets are quantitatively illustrated in Figure 3, with the respective peptide sequence information being described in Supplementary Table S1. For each dataset, the top-five variables per PLS component are described below. For the clinical dataset, the top variables affecting this reduced space were serum urea, vitamins, blood substitutes and perfusion solutions, antigout preparations, beta-blocking agents, total blood protein, agents acting on the renin–angiotensin system, Bristol stool score, sex hormones and modulators, haemoglobin, and urinary erythrocytes (10^6^/L). For the microbiome dataset, these were microbes from the taxa *Roseburia*, *Bacteroidetes*, *Holdemania*, *Ruminococcus2*, *Bacteria*, *Veillonella*, *Allisonella*, *Haemophilus*, *Adlercreutzia*, *Senegalimassilia*, *Dialister*, and *Slackia.* Combining the clinical and microbiome datasets, the following proved to be the most impactful: serum urea, vitamins, blood substitutes and perfusion solutions, antigout preparations, *Howardella*, total blood protein, *Oligosphaera*, *Anaerofustis*, *Cloacibacillus*, *Enterorhabdus*, drugs used in diabetes, *Senegalimassilia*, and *Dorea.* On the other hand, the most impactful peptides were derived from parental proteins such as collagen alpha-1(I) chain (COL1A1), collagen alpha-1(II) chain (COL2A1), collagen alpha-2(I) chain (COL1A2), collagen alpha-1(XVI) chain (COL16A1), collagen alpha-1(III) chain (COL3A1), CD99 antigen (CD99), keratin, type I cytoskeletal 10 (KRT10), and fibrinogen alpha chain (FGA). When the clinical variables were added to the peptidome dataset, the most impactful variables were related to serum urea, COL1A1, COL2A1, COL1A2, COL16A1, COL3A1, CD99, FGA, and KRT10. When instead of the clinical dataset, the microbiome dataset was combined with the peptidome dataset, then the largest impact on the dataspace was observed from microbes and fragments from the taxa and parental proteins, respectively, COL1A1, COL2A1, COL1A2, COL16A1, COL3A1, CD99, FGA, and *Enterorhabdus*. Combining all datasets, the list with the most impactful variables included serum urea, COL1A1, COL2A1, COL1A2, COL16A1, COL3A1, CD99, and FGA.

### 3.4. Visualization of the Matched Participants in the 3D Space

To account for the impact of the clinical confounders, the urinary peptidome of a historical cohort (n = 408) in which the four groups were equally numbered and matched for the clinical characteristics presented in Table 1 was considered. The preprocessing part of the presented pipeline was applied again, this time considering the same 812 peptides that remained after applying the pipeline to the urinary peptidome of the original cohort (n = 110). Following the PLS feature extraction, the matched participants were visualized in the 3D space (Supplementary Figure S3) to explore whether members of the same disease state group would be proximally positioned. Along these lines, although a degree of overlap was observed (predominantly regarding the Moderate CKD group), participants still displayed formations similar to the ones displayed by the original cohort (Supplementary Figure S1D).

## 4. Discussion

The human urinary peptidome and fecal microbiome, although promising, have not yet been integrated to cluster individuals in the CKD context. In this study, we attempted, for the first time, a multidisciplinary approach that involved clinical, fecal microbiome, and urinary peptidome data in a cohort of 110 CKD and Non-CKD participants. Individuals were grouped as Non-CKD or CKD, with the latter being labeled as Early, Moderate, and Advanced based on eGFR values. The aim was to explore the capacity for cohort separation and distinct cluster formation based on the aforementioned disease state groups. Along these lines, direct comparisons were performed using these three datasets alone or in combinations. Each time, participants were visualized as single data points in a 3D space, and the observed disease state formations were assessed. Clustering was also applied, inspecting the correspondent concordance between cluster content and disease states. Lastly, the impact of each variable in the reduced dataspace was examined to determine potential underlying connections with disease pathophysiology. 

Several remarks can be made for the cohort visualizations in the 3D space (Supplementary Figure S1A–G) and the eGFR-based clustering results (Figure 2). To begin with, the capacity of the fecal microbiome dataset for distinct cluster formation appeared lower than that of the urinary peptidome dataset, the latter demonstrating superior cohort separation along with higher cluster purity and silhouette values. Although both data sources are from non-invasive sample collections and capable of captivating systemic/peripheral changes, urine (in addition to being easier and more straightforward to obtain) is more proximal to the kidney system, thus potentially better reflecting disease state. Further, regarding technical detection, peptides are considered more stable than RNA in terms of both temperature and cleavage enzymes. On the other hand, the microbiome’s capacity was substantially boosted by adding it to the clinical dataset, while this appeared to be enhanced to a lesser degree in the case of the peptidome. This might be attributed to the numerous clinical confounders of the microbiome [49]. Nevertheless, combining the clinical and microbiome datasets did not diminish the overlap between the Moderate and Early CKD groups. On the other hand, the presence of the peptidome improved the plots along these lines. This observation, along with the fact that all plots generated by the peptidome (alone or in combination with the other datasets) were similar, is indicative of the magnitude of the variation that was captured by this omics dataset. Particularly, participant visualization in the 3D space using solely the urinary peptidome was repeated using a historical cohort almost four times larger than the original one,generated after matching for theseveral main clinical confounders. Although separation in this matched and larger cohort was still evident, it was less efficient than before (especially due to overlaps regarding the Moderate CKD group). This potentially indicates that the key to maximum separation is including clinical information along with molecular data. This should be further explored in future work. Further, the disease state continuum and the distant and relatively localized Non-CKD group (with the exception of the microbiome plot), as well as the fact that the 3D plots, cluster purity, and silhouette values were generally in line, add additional validity to our findings.

Another aspect that might require an in-depth investigation is the unconventional position of some patients in these plots. For instance, in Supplementary Figure S1F (microbiome and peptidome), a patient (PLS1: +12.5, PLS2: −5.4, PLS3: +2.9) with Moderate CKD and eGFR 33 mL/min/1.73 m^2^ is located towards the Advanced CKD formation, presumably due to being near the latter group’s eGFR threshold. Nevertheless, it is not apparently clear why that patient is almost antidiametric to another one (PLS1: −1.2, PLS2: +11.2, PLS3: +2.9) of the same group, even though they have similar kidney function (eGFR 30 mL/min/1.73 m^2^). Unexpected positioning examples regarding the Moderate CKD group’s upper eGFR threshold can also be observed (e.g., patients PLS1: +11.4, PLS2: +0.1, PLS3: +0.9 with eGFR 55 mL/min/1.73 m^2^; and PLS1: −9.6, PLS2: +8.1, PLS3: +3.9 with eGFR 59 mL/min/1.73 m^2^). Interestingly, these relative positions are also maintained in the respective combined clinical, microbiome, and peptidome plot (Supplementary Figure S1G). This unexpected positioning could be attributed to potential similarities in molecular profiles favoring the proximal positioning of participants who, nevertheless, display to some degree differences in the eGFR. In the past, patients with similar kidney function have been described as displaying differences in their molecular profiles [50]. Thus, it should be further explored whether this positioning is an indication that the molecular profile of an individual reflects an underlying disease property (e.g., risk of progression or cardiovascular outcome) that could go undetected by the current gold clinical standards. This would require to be studied in-depth in the future with close follow-up, after initially examining how other factors such as a shared CKD aetiology, affect the positioning of the participants.

As expected, among the most impactful clinical variables (Supplementary Figure S2A,C,E,G) were serum urea and haemoglobin, urinary albumin, total blood protein and urinary erythrocytes, as well as blood pressure and cholesterol measures, smoking, BMI, and Bristol stool score. Included in this list are medications targeting hypertension, such as beta-blocking agents, calcium channel blockers, and agents that act on the renin–angiotensin system. This inclusion is attributed to the significance of effectively managing hypertension in CKD [51]. 

Several of the most impactful microbiota presented (Supplementary Figure S2B,C,F,G) have also been highlighted in the CKD literature. For example, the genus *Roseburia* had the lowest levels in the Advanced (stage 5) CKD patients [10]. In the same study, the genus *Flavonifractor* had higher levels in CKD vs. Non-CKD individuals. In another study, the CKD vs. Non-CKD group was found to be deprived of bacteria of the *Burkholderiales* order [52]. The authors also described, within a linear discriminant analysis, an enrichment of the genus *Holdemania* in CKD. Investigating IgAN, patients had lower levels of microbiota belonging to the *Synergistaceae* family than Non-CKD participants [53]. Further, the genus *Enterorhabdus* has been negatively correlated with IL-10 and IL-4 inflammation factors [54], while the genus *Butyricicoccus* has been positively correlated with eGFR [55]. In a Mendelian randomization study, the genus *Senegalimassilia* was found relevant in the context of CKD [56]. Further, a species of the genus *Haemophilus* displayed an increased trend in CKD compared to Non-CKD participants [57]. Another example is the *Veillonella* genus, which was present at different levels in a mixed Moderate/Advanced CKD group vs. a Non-CKD group as well as an Early CKD group [58]. Several gut bacteria species belonging to genera such as *Roseburia*, *Butyricicoccus*, or *Ruminococcus* have the potential to produce short-chain fatty acids (SCFAs) like acetate, propionate, and butyrate [59]. A variety of these SCFAs have the capacity for receptor binding, thus having modulatory potential in, e.g., inflammation and oxidative stress, autophagy, energy metabolism, and immune pathways [60]. Despite the fact that a number of factors appear to influence the gut microbiome [49], inevitably introducing variability, especially in several relatively low-size studies in this relatively new field, a noteworthy common ground is the fact that the CKD pathophysiological environment appears to impede the growth of beneficial SCFA-producing microbiota, thus consequently depriving CKD patients of the blessings of their metabolites.

The top 20 peptidome variables in all different datasets (Supplementary Figure S2D,E,F,G) have been derived from 16 parental proteins. In more detail, these fragments originated from collagen proteins, namely, COL1A1, COL2A1, COL3A1, COL16A1, collagen alpha-1(XIX) chain (COL19A1), COL1A2, collagen alpha-2(IV) chain (COL4A2), and collagen alpha-2(V) chain (COL5A2), but also from non-collagen proteins, including apolipoprotein A-I (APOA1), CD99, cadherin-related family member 5 (CDHR5), FGA, sodium/potassium-transporting ATPase subunit gamma (FXYD2), KRT10, vesicular integral-membrane protein VIP36 (LMAN2), and matrix Gla protein (MGP). Nevertheless, it was from collagen that the majority of these peptides were derived. Collagen proteins are amongst the main components of the extracellular matrix, which is accumulated in the kidney during the fibrotic phenomenon [61], which is a hallmark of CKD. Fragments from all of these proteins except CDHR were among the significantly different findings when comparing a matched cohort of Early (stage 2) vs. mixed Moderate/Advanced (stages 3b−5) CKD patients [12]. In detail, out of the 51 (48 unique) peptides presented in Supplementary Figure S2G (clinical, microbiome, and peptidome dataspace), 38 were found to be significantly different in the aforementioned comparison, as well as 2 when comparing fast and slow progressors within the Early CKD group.

The number of impactful variables per type (clinical, microbiome, peptidome) in the combined datasets (Supplementary Figure S2C,E,F,G) was in line with the 3D visualizations and clustering results. To illustrate, the aforementioned boost for cohort separation after the addition of the clinical dataset in the microbiome dataset is reflected by the numerous impactful clinical variables (53%) regarding the respective reduced dataspace (Supplementary Figure S2C). In contrast, after the addition of clinical variables to the peptidome dataset, although serum urea was again the most impactful variable on the first PLS component, collectively, the total number of impactful clinical variables was low (3%) (Supplementary Figure S2E). Similarly, low numbers of impactful non-peptidome variables were recorded after the addition of the microbiome (8%) (Supplementary Figure S2F) or both the clinical and microbiome variables (3% and 12%, respectively) (Supplementary Figure S2G) in the peptidome dataset. Further, in the presence of the urinary peptidome dataset, specific peptides consistently were proved impactful. These observations, although notably made in this small cohort, might be indicative of the substantial variation captured by the urinary peptidome in CKD. However, it is noteworthy to mention that certain relevant clinical variables, such as age or serum creatinine, were excluded by design from the analysis due to their relation to the eGFR [18].

Our study has several limitations. First of all, although a variety of clinical data were included, information on several confounders (expected to especially affect the fecal microbiome) was not available. For example, data regarding exercise, diet, or country of origin were missing. It is worth noting, however, that participants were recruited from the same hospital in Ghent, and CKD patients are expected, to a degree, to be encouraged to follow common diet patterns, such as avoiding foods that would lead to hyperkalemia [62]. Further, shotgun metagenomics is expected to be more informative than the amplicon sequencing of the fecal microbiome. In addition, since a standard preprocessing protocol is not yet fully established in the microbiome field, common methods (e.g., proportion-based normalization) were followed across our pipeline. Last, caution should be taken not to confuse any here-presented correlational relationship as causational. Thus, given the relatively low sample size and imbalanced disease state groups, more extensive studies are warranted to validate and further expand the presented results involving multiple ethnicities or geographical origins and designed toward utilizing as many clinical confounders as possible. Additionally, future studies should assess the effects of missing value imputation and dimensionality reduction methods, encompassing the chosen dimensions and target label, along with various clustering methods and quality metrics.

At the same time, this study has several strengths. To our knowledge, this is the first time that an attempt has been made to directly evaluate the fecal microbiome and the human naturally occurring urinary peptidome in terms of their capacity for capturing variation in CKD as standalone datasets or in combinations. In addition, a large part of microbiome CKD studies focus on patients with kidney failure, while the investigated cohort covered patients across the entire CKD stage spectrum and not undergoing dialysis. Importantly, the presented preprocessing pipeline can be implemented in both clinical and (even multiple different) omics datasets, towards maximizing their combined utility in analyses. This is highly relevant in machine learning processes, in which several algorithms cannot handle categorical data, e.g., support vector machines. That said, often carefully designed and promising models in the literature, rely solely on omics data, considering clinical variables only for establishing cohort exclusion criteria. In that way, not only is a substantial part of potentially valuable clinical information not utilized, but also the applicability of the developed models is isolated only to a subset of the patient population. Thus, parts of the presented pipeline may as well be utilized towards enhancing model development in the biomedical field with the ultimate goal of clinical practice implementation.

## 5. Conclusions

The urinary peptidome appears to capture a substantial part of the variation in CKD, mainly with collagen but also several non-collagen parental proteins. When combining both clinical and molecular information, an improved cohort separation was observed compared to when using clinical data alone. Such a combination is expected to boost the performance of (often single omics-based) machine learning models designed to support clinical practice. The necessity for validation in broader, more comprehensive studies involving, among others, shotgun sequencing microbiome data, an expanded array of clinical data, and alternative clustering methods or even CKD grouping is underscored. Future studies are also encouraged to have as their main focus the exploration of potential bacterial proteins in urine and their association with gut bacteria and the gut proteome based on pathway analyses. Studying cohort separation through the lens of clinical outcomes might also shed more light on why patients with similar clinical characteristics appear to have different (e.g., kidney and cardiovascular) disease course.

## Figures and Tables

**Table 1:** Original and matched historical cohort clinical characteristics.

Characteristics	Non-CKD	Early CKD	Moderate CKD	Advanced CKD	*p-*Value *
(A) Original cohort.					
n	10	32	40	28	
Age	41.5 (13.1)	52.1 (16.7)	63.6 (14.8)	68.9 (13.5)	<0.001
BMI	22.4 (3.3)	25.7 (3.8)	27.8 (5.1)	27.1 (3.6)	0.004
eGFR	79.3 (19.0)	88.7 (22.5)	44.3 (9.7)	19.1 (6.6)	<0.001
Systolic blood pressure	118.4 (27.9)	130.0 (16.0)	137.1 (19.1)	145.2 (21.7)	0.002
Diastolic blood pressure	70.3 (9.2)	79.8 (11.8)	79.9 (12.2)	79.1 (8.6)	0.121
Female	7 (70.0)	14 (43.8)	14 (35.0)	6 (21.4)	0.041
(B) Matched historical cohort.					
n	102	102	102	102	
Age	72.3 (4.2)	72.4 (8.8)	72.4 (8.0)	74.6 (7.9)	0.085
BMI	29.5 (6.0)	29.8 (5.9)	30.2 (5.7)	31.3 (6.6)	0.152
eGFR	96.6 (8.9)	79.6 (16.6)	45.4 (8.9)	22.9 (5.6)	<0.001
Systolic blood pressure	138.8 (13.4)	142.8 (18.1)	139.9 (21.4)	139.8 (20.7)	0.469
Diastolic blood pressure	76.8 (8.2)	75.4 (9.6)	74.5 (12.6)	77.3 (10.8)	0.205
Female	35 (34.3)	35 (34.3)	32 (31.4)	29 (28.4)	0.774

In the table, n refers to the number of subjects per group. Available clinical information was used to calculate the mean (SD) and number (percentage) for numeric and categorical variables, respectively. SD: standard deviation; BMI: body mass index; eGFR: estimated glomerular filtration rate. * *p*-values are based on analysis of variance (ANOVA).

## Data Availability

The availability of the clinical and microbiome data is described in the respective original publications. Peptidome data will be provided by the corresponding author, depending on scientific merit, after a data access and confidentiality agreement is signed.

## Supplementary Materials

The following supporting information can be downloaded at: https://www.mdpi.com/article/10.3390/proteomes12020011/s1. Table S1: Urinary peptidome data. Information on the (raw) relative abundance of the sequenced 5071 peptides (columns) for participants (rows) belonging to the original (n = 110) and matched historical (n = 408) cohorts is provided. Given is also the sequence information regarding the peptides that are among the top 20 most impactful variables per PLS component of the clinical, microbiome, and peptidome dataspace. Figure S1A–G: Cohort visualizations in the 3D space per dataset. Each single dot represents an individual labeled with the respective eGFR. The color is based on the corresponding disease state, namely, Non-CKD (blue), Early (yellow), Moderate (orange), and Advanced (red) CKD stages. CKD: chronic kidney disease; eGFR: estimated glomerular filtration rate. Figure S2: PLS loadings. The top 20 most impactful variables per PLS component are shown for each dataset. The X axis refers to the PLS coefficient value. In the Y axis, variables of the urinary peptidome are labeled with their parental protein name and corresponding amino acid position (peptide ID in parenthesis), while those of the fecal microbiome are labeled using the relevant taxa information. Colors indicate that a variable is either clinical (teal), microbiome (magenta), or peptidome (gold). APOA1: apolipoprotein A-I; CD99: CD99 antigen; CDHR5: cadherin-related family member 5; COL16A1: collagen alpha-1(XVI) chain; COL19A1: collagen alpha-1(XIX) chain; COL1A1: collagen alpha-1(I) chain; COL1A2: collagen alpha-2(I) chain; COL2A1: collagen alpha-1(II) chain; COL3A1: collagen alpha-1(III) chain; COL4A2: collagen alpha-2(IV) chain; COL5A2: collagen alpha-2(V) chain; FGA: fibrinogen alpha chain; FXYD2: sodium/potassium-transporting ATPase subunit gamma; KRT10: keratin, type I cytoskeletal 10; LMAN2: vesicular integral-membrane protein VIP36; MGP: matrix Gla protein. Figure S3: Matched cohort visualization in the 3D space.
